# Disability, noncommunicable disease and health information

**DOI:** 10.2471/BLT.15.156869

**Published:** 2016-02-03

**Authors:** Nicola C Richards, Hebe N Gouda, Jo Durham, Rasika Rampatige, Anna Rodney, Maxine Whittaker

**Affiliations:** a School of Public Health, University of Queensland, 288 Herston Road, Herston, QLD 4006, Australia.

Noncommunicable diseases (NCDs) are a major cause of preventable disability worldwide.^1^ While actions to monitor NCDs have gained significant momentum in the global health agenda, similar developments to monitor and manage the growing burden of NCD-related disability have been relatively slow. The global NCD action plan was developed to support country efforts in addressing the devastating social, economic and public health impacts of NCDs.^2^ The NCD action plan includes nine voluntary targets and a monitoring framework. However, the monitoring framework has been criticized for its focus on mortality while neglecting adequate measures of morbidity and disability.^3^ This has resulted from focusing on existing data within health information systems, rather than on identifying appropriate data for measuring disease burden. Indeed, the issue of the chronicity of NCDs seems absent in most monitoring and evaluation frameworks, with an implicit assumption that the only outcome of interest for countries is premature mortality.

Responding to this limited scope, several alternative frameworks have been proposed to provide more comprehensive approaches to the prevention and control of NCDs. While these frameworks offer a broader approach to NCD prevention, they offer little for the millions of people already living with an NCD. Disability related to NCDs needs to be addressed in line with principles of the Convention on the Rights of Persons with Disabilities, including the provision of long-term rehabilitative services. As defined in the Convention, persons with disabilities are those who have long-term physical, mental, intellectual or sensory impairments, which hinder their full and effective participation in society on an equal basis with others. Here we argue for the need to improve health data by incorporating measures of NCD-related disability into discussions on NCD prevention and control; strengthen information systems to better capture data on disability; and to align NCD and disability measurement and monitoring strategies.

**Fig. 1:**
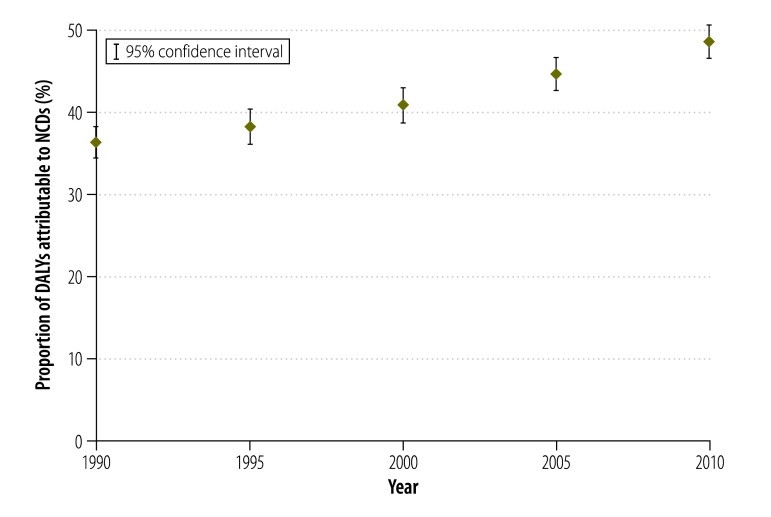
The proportion of disability-adjusted life years attributable to noncommunicable disease: low- and middle-income countries, 1990–2010

Low- and middle-income countries have a large and growing NCD burden (Fig. 1). NCDs already account for two out of every three years lived with a disability^5^ and in 2008, 80% (29 million) of all NCD-related deaths were in low- and middle-income countries.^5^ NCDs also affect people at younger ages in low- and middle-income countries, with around one-third of NCD-related deaths occurring among people younger than 60 years of age.^1^ Seventy per cent of people living with diabetes live in low- and middle-income countries,^6^ where access to health care and social support are limited. It is estimated that between 2008 and 2030, diabetes, cardiovascular diseases, cancer, chronic respiratory diseases and mental illnesses will have cost low- and middle-income countries 21 trillion United States dollars due to illness and lost production.^1^ Disability also contributes to higher NCD risk through an increased risk of sedentary lifestyles, producing a vicious cycle between NCDs and disability.^7^

DALY, disability-adjusted life year; NCD: noncommunicable disease.

Source: Institute for Health Metrics and Evaluation.^4^

The four largest contributors to NCD-related morbidity and mortality (cardiovascular diseases, chronic respiratory diseases, cancer and diabetes), can lead to impairments related to amputations, blindness, mobility and speech. Such disabilities can reduce productivity, increase demand on the social and health systems and impoverish families.^5^ While limited, the available data suggests that in low- and middle-income countries, people with disabilities experience worse socioeconomic and health outcomes than their able-bodied counterparts. People with disabilities in Asia and the Pacific are among the poorest and most marginalized in the region, with comparatively lower educational attainment, lower employment and poorer standards of living and health outcomes.^8^ The chronic disabling states caused by NCDs are a significant component in this global public health issue^7^ and need to be recognized and incorporated in all global action and monitoring frameworks on NCD prevention, control and treatment.

Statistics on causes-of-death are absent for around 85% of the worlds’ population (65 primarily low- and middle-income countries), and data on risk factors are equally lacking.^9^ It is not surprising therefore that information on the prevalence of NCD-related disability and corresponding rehabilitation needs are scarce. This however, is despite the stated priorities of the global NCD action plan, which are to “reduce the preventable and avoidable burden of morbidity, mortality and disability due to NCDs”.^5^ There is nothing in the plan or global monitoring framework relating to monitoring measures of disability or rehabilitation, despite initially stating that rehabilitation needs to be a central health strategy in NCD programmes, both to address risk factors (such as resulting physical inactivity) or loss of function due to NCDs (for example, from blindness).

The global prevalence of disability in low- and middle-income countries has been estimated. However, these estimates are imprecise, based on census, survey and registration information and the epidemiology of specific conditions.^7^ For many low- and middle-income countries, the Global Burden of Disease study estimates of disability-adjusted life years (DALYs) are all that is available. While this may provide an interim solution, incomplete data mean that the estimates are derived from several complex steps resulting in considerable uncertainty. Further, the DALY assumes a particular medical condition has the same impact on a person’s years lived with a disability, irrespective of the context in which they live. The international classification of functioning, disability and health, refers to limitations people with disabilities actually experience.^10^

To obtain a more accurate picture of disability in low- and middle-income countries, existing disability estimation techniques need to be further developed and improved.^11^ This requires local valid data on rates of NCD-related disability, statistics on functional status, rehabilitation needs, and the coverage and utilization of relevant health services. The required data range from simple counts to disaggregated indicators, and to complex comparisons of quality of life and social participation for those with and without disability. With such data, disease burden can be measured, the effectiveness of health interventions can be evaluated and planning decisions can be made. Many countries will struggle to collect data on disability, but while it is important to be pragmatic about country capacities, it is also essential that we avoid skewing global efforts and neglecting the significant and growing burden of NCD-related disability.

Actions to address major NCDs have focused on prevention efforts through the modification of risk factors. As a result, most action plans and corresponding monitoring and evaluation frameworks are limited to measures of risk factor prevalence.^12^ Capacity to respond to NCDs, morbidity, disability and premature deaths were acknowledged as some of the most significant issues facing the WHO Eastern Mediterranean Region.^13^ However when discussing monitoring frameworks, measures of disability are absent; focusing instead on risk factors, morbidity and mortality and health system capacity to respond.^13^

The primary goals of NCD care are to enhance functional status, minimize symptoms and prolong and enhance quality of life, rather than to cure.^1^ These outcomes are not captured by current episode-based health information systems. Improved information systems must extend beyond single risk factors and disease-based perspectives, to create surveillance systems that include the whole lifespan.^14^ This will require a shift in health policies and systems, including the capacity to measure improvements in health outcomes across multiple conditions, stronger disability and rehabilitation information systems and better coordination with disability services.^1^

In 2011, the *World report on disability* recommended improving national disability statistics and the comparability of data, developing appropriate tools and filling research gaps, increasing research and promoting evidence-based practice for rehabilitation services.^15^ The international classification of functioning, disability and health provides universal and consistent language to describe disability and is consistent with the social model of disability in the Convention on the Rights of Persons with Disabilities.

Information on NCD-related disability should form a vital component of NCD policy and planning to fulfil the rehabilitation aspects of the Convention and identify cost-effective approaches to rehabilitation services and positive health outcomes. Including a disability perspective in discussions on NCD prevention, control, treatment and measurement is in line with the sustainable development goals, which aim to ensure healthy lives and well-being, while reducing inequality within and among countries. These recommendations must be integrated into NCD policies and national information systems through linkages to appropriate data sources. Data on functional limitations, including physical activities and social participation, must be included and collected among people living with NCD-related disabilities. Without such information, the development of meaningful measures of NCD-related disability will remain elusive.
